# High cell density cultivation and recombinant protein production with *Escherichia coli *in a rocking-motion-type bioreactor

**DOI:** 10.1186/1475-2859-9-42

**Published:** 2010-05-30

**Authors:** Julia Glazyrina, Eva-Maria Materne, Thomas Dreher, Dirk Storm, Stefan Junne, Thorsten Adams, Gerhard Greller, Peter Neubauer

**Affiliations:** 1 Laboratory of Bioprocess Engineering, Department of Biotechnology, Technische Universität Berlin, Ackerstraße 71-76, D-13355 Berlin, Germany; 2 Sartorius Stedim Biotech GmbH, August Spindler Str. 11, D-37079 Göttingen, Germany

## Abstract

**Background:**

Single-use rocking-motion-type bag bioreactors provide advantages compared to standard stirred tank bioreactors by decreased contamination risks, reduction of cleaning and sterilization time, lower investment costs, and simple and cheaper validation. Currently, they are widely used for cell cultures although their use for small and medium scale production of recombinant proteins with microbial hosts might be very attractive. However, the utilization of rocking- or wave-induced motion-type bioreactors for fast growing aerobic microbes is limited because of their lower oxygen mass transfer rate. A conventional approach to reduce the oxygen demand of a culture is the fed-batch technology. New developments, such as the BIOSTAT^® ^CultiBag RM system pave the way for applying advanced fed-batch control strategies also in rocking-motion-type bioreactors. Alternatively, internal substrate delivery systems such as EnBase^® ^Flo provide an opportunity for adopting simple to use fed-batch-type strategies to shaken cultures. Here, we investigate the possibilities which both strategies offer in view of high cell density cultivation of *E. coli *and recombinant protein production.

**Results:**

Cultivation of *E. coli *in the BIOSTAT^® ^CultiBag RM system in a conventional batch mode without control yielded an optical density (OD_600_) of 3 to 4 which is comparable to shake flasks. The culture runs into oxygen limitation. In a glucose limited fed-batch culture with an exponential feed and oxygen pulsing, the culture grew fully aerobically to an OD_600 _of 60 (20 g L^-1 ^cell dry weight). By the use of an internal controlled glucose delivery system, EnBase^® ^Flo, OD_600 _of 30 (10 g L^-1 ^cell dry weight) is obtained without the demand of computer controlled external nutrient supply. EnBase^® ^Flo also worked well in the CultiBag RM system with a recombinant *E. coli *RB791 strain expressing a heterologous alcohol dehydrogenase (ADH) to very high levels, indicating that the enzyme based feed supply strategy functions well for recombinant protein production also in a rocking-motion-type bioreactor.

**Conclusions:**

Rocking-motion-type bioreactors may provide an interesting alternative to standard cultivation in bioreactors for cultivation of bacteria and recombinant protein production. The BIOSTAT^® ^Cultibag RM system with the single-use sensors and advanced control system paves the way for the fed-batch technology also to rocking-motion-type bioreactors. It is possible to reach cell densities which are far above shake flasks and typical for stirred tank reactors with the improved oxygen transfer rate. For more simple applications the EnBase^® ^Flo method offers an easy and robust solution for rocking-motion-systems which do not have such advanced control possibilities.

## Background

Disposable cultivation technologies are an innovative alternative to traditional reusable bioreactor systems. They offer advantages to the biopharmaceutical industry such as manufacturing flexibility, simplicity of operation, decreased incidence of contamination, and significantly lower costs for cleaning, sterilization and validation [1]. Therefore, the utilization of disposable bioreactors in manufacturing processes strongly increased during the last ten years, especially in the area of mammalian cell culture for production of high value biopharmaceuticals. Concerning mass and energy transfer, these bioreactors can be classified into the following groups: static bag bioreactors, mechanically driven bag bioreactors (with stirrer, vibromixer or wave-induced motion), pneumatic driven bioreactors (bubble column, airlift reactors) and hybrid bag bioreactors, where mechanical and pneumatic power inputs are combined [1,2]. The characteristics of these different types of bioreactors have been thoroughly reviewed in a number of recent papers [1-5]. Therefore we will focus here on the use of rocking-motion-type bag bioreactors which were introduced into the market during late 1990s [6]. These systems consist mainly of a reservoir, typically a bag, made of polymeric materials such as polyethylene, polystyrene, polytetrafluorethylene, or polypropylene. The pre-sterilized and assembled cultivation chamber is situated on a rocking platform that induces wave motion to the culture fluid for mixing and bubble-free oxygen transfer.

Hydrodynamic and oxygen transfer studies of rocking-motion-type bioreactors and comparisons with conventional stirred cell culture bioreactors are described in literature and have been recently thoroughly reviewed [4]. Under optimal conditions, the oxygen transfer reached comparable or higher values to those in stirred cell culture bioreactors with membrane or surface aeration. A number of authors measured *k_L_a *values in rocking-motion-type bioreactors which generally lay between 4 and 20 h^-1 ^depending on rocking angle, rocking rate, bag geometry, culture volume, and gas composition [4]. The impacts of rocking rate and angle are much higher than of the gas composition, clearly indicating the limitation in gas transfer. Improvements by sparging systems, aeration membranes or baffles helped to obtain higher K_L_a values up to 80 h^-1^. Highest K_L_a values (> 700 h^-1^) have been reported for the CELL-trainer^® ^which is characterized by an additional horizontal displacement (reviewed in [1,4]).

Rocking-motion-type bioreactors are most widely used with animal and plant cells, mainly for the production of recombinant proteins in insect cells [7], monoclonal antibodies in animal cells [8-11], baculovirus in insect cells [12], and for several proteins and secondary metabolites, such as ginsenosides in plant cell cultures [13,14]. In contrast the results with bacterial cells with rocking-motion-type bioreactors are few and surprisingly low cell densities are obtained. Only recently, Eibl et al. reported cell densities of 1 × 10^9 ^cells per ml, corresponding to about 0.5 g L^-1 ^cell dry weight, of *E. coli *in a GMP process with a BioWave^® ^system [4]. These cell densities are about 2 log-orders of magnitude lower compared to typical high cell density processes with *E. coli*.

The main disadvantage of typical surface-aerated rocking-motion-type bioreactors for the cultivation of aerobic microbes is the low oxygen transfer rate compared to stirred tank bioreactors with air sparging systems.

In particular, microbial high cell density cultures have a high metabolic oxygen demand. In these cultures the oxygen transfer rate of the bioreactor determines the maximum biomass concentration. Unfortunately, the solubility of oxygen is even decreasing with increasing cell densities due to a higher viscosity of the cell suspension. Therefore, in order to enhance the existing oxygen transfer limitation for aerobic high cell density cultivation in disposable bioreactors, an aeration membrane or disposable spargers and baffles have been inserted [4] or the medium was vibrated [15].

There are very few published attempts to cultivate aerobic microbes in rocking-motion-type bioreactors. One report presents the cultivation of yeast *Saccharomyces cerevisiae *in a wave-motion-type bioreactor which was modified with a frit sparger [16]. The final dry cell weight was about 9 g L^-1 ^which was almost two times higher compared to shake flask cultures. Also the maximum specific growth rate and biomass yield were increased. However, despite the relatively high cell density, the sparger in the bag was ineffective in increasing the oxygen transfer capacity; cultures of *S. cerevisiae *grew equally with oxygen blending into the air stream in bags with the sparger compared to standard bags. k_L_a values of 38 h^-1 ^with air or of 60 h^-1 ^with oxygen-blended air were obtained, which is still at least an order of magnitude below the oxygen transfer coefficients of laboratory stirred tank reactors.

Another report describes the growth of *Corynebacterium diptheria *for vaccine production in the CultiBag RM system [17]. At *k_L_a *values of 6.0 h^-1^, a final cell density of OD_590 _= 5 compared to OD_590 _= 7.3 in an aerated stirred tank bioreactor was achieved. However, compared to the cultivation of this slowly growing bacterium, the aerobic cultivation with *E. coli *would demand higher oxygen transfer rates.

Oxygen limitation in cultures which aim for high cell densities is generally circumvented by applying the fed-batch cultivation mode. The oxygen uptake rate of a culture correlates with the carbon substrate consumption rate. Therefore, it can be controlled by continuous growth-limiting addition of the carbon substrate to the culture. Additionally, the carbon limited fed-batch cultivation provides further benefits by the possibility to avoid overflow metabolism and overflow related medium acidification [18].

Recently a simple-to-use cultivation technology has been introduced which extends the advantages of the fed-batch principle to shaken cultures, where external feeding is difficult to achieve. The EnBase^® ^platform applies an internal delivery of glucose by biocatalytic degradation of glucose containing polymers [19]. Thus no external pump is necessary and the system is applicable in shaken culture formats. A further development of the method from the initially introduced gel based two-compartment system to entire liquid formulations, named EnBase^® ^Flo, provides a higher flexibility; it can be applied in systems with optical sensors and enables scale up [18]. In Enbase^®^, cell growth and oxygen consumption is controlled by the amount of a biocatalyst, similar like the pump rate is adapted in a conventional fed-batch system. The release of glucose by the biocatalyst with a constant rate yields a quasi linear biomass increase, and consequently an approximately stable DOT. Simply by enhancing the biocatalyst, i.e. the glucose release rate, such cultures can be optimized to grow at the limit of the oxygen transfer capacity in the system, corresponding to the maximum possible volumetric growth rate. The controlled growth also reduces side metabolite production and thus provides a stable pH in the culture. Further, optimization of trace elements [20] and addition of complex additives [18] increased the robustness of the system in view of oxygen limitation, expression systems, and target proteins. We suggested that the application of EnBase^® ^Flo would also simplify the cultivation process and improve the product yield in rocking-motion-type bioreactors.

In this study we demonstrate the possibility of obtaining high cell densities of the bacterium *E. coli *in the BIOSTAT^® ^CultiBag RM system either by a glucose limited fed-batch procedure or by the Enbase^® ^Flo technique. Furthermore, we demonstrate at one example, a heterologous alcohol dehydrogenase (ADH) produced in *E. coli*, that the system can be successfully applied for recombinant protein production.

## Methods

### Bacterial strains

E. coli BL21 and BL21(DE3) (Novagen, Merck KgaA, Darmstadt, Germany) and the recombinant strain *E. coli *RB791 pAdh [21] encoding a heterologous alcohol dehydrogenase were applied in this project.

### Batch and fed-batch cultures

#### Medium composition

The mineral salt medium ([22], modified) contained Na_2_HPO_4 _(8.6 g L^-1^), KH_2_PO_4 _(3 g L^-1^), NH_4_Cl (1 g L^-1^), NaCl (0.05 g L^-1^) and glucose (10 g L^-1^). Glucose was autoclaved separately. A sterile trace element solution was added to supply the medium with (final concentration) CoCl_2 _× 6 H_2_O (0.25 mg L^-1^), CuCl_2 _× 2 H_2_O (0.15 mg L^-1^), H_3_BO_3 _(0.3 mg L^-1^), Na_2_MoO_4 _× 2 H_2_O (0.25 mg L^-1^), Zn(CH_3_COO)_2 _× 2 H_2_O (0.8 mg L^-1^), Titriplex III (0.84 m g L^-1^), Fe(III) citrate (6 mg L^-1^) and MnCl_2 _× 2 H_2_O (1.23 mg L^-1^). Furthermore, MgSO_4 _× 7 H_2_O (0.5 g L^-1^) was added. The feeding solution, for the fed-batch cultivation, contained glucose (660 g L^-1^) and MgSO_4 _× 7 H_2_O (19.7 g L^-1^).

#### Shake flask cultures

Shake flask cultures were performed in glucose-based mineral salt medium in Sensolux-EF 250 mL flasks (Sartorius) with a filling volume of 75 mL. The flasks were cultivated in a Certomat T plus shaker (Sartorius AG, Göttingen, Germany) with a displacement of 5 cm at 200 rpm and 37°C.

#### Cultivation setup

Precultures were grown in 50 ml Sartorius CultiFlasks, with 20 ml working volume. They were incubated for at least 6 h at 37°C and 130 to 150 rpm.

The main batch cultures were performed in a BIOSTAT^® ^CultiBag RM 20 system with a 10 L bag without sensors containing a culture volume of 1 L. The rocking angle was set to the maximum tilt level of 10°, the rocking rate was manually controlled between 35 rocks min^-1 ^and the maximum (42 rocks min^-1^). The temperature was set to 25°C or 37°C as indicated in each experiment. The airflow was regulated between 1 and 6 L min^-1^.

Fed-batch cultivations were performed in a BIOSTAT^® ^CultiBag RM 20 optical system using a CultiBag RM 10 L bag with a culture volume of 4 L and optical sensors. The rocking angle was set to the maximum tilt level of 10°, the rocking rate was set to 35 rocks min^-1 ^and increased during the cultivation to a maximum of 42 rocks min^-1^, the temperature was adjusted to 25°C and later increased to 37°C. The airflow was set to 1 L min^-1 ^and later increased to 6 L min^-1^.

#### Analytical methods

Growth of the cultures was followed by measuring the light absorbance at 600 nm (OD_600_). The glucose concentration was determined by a glucose test kit (R-Biopharm AG, Darmstadt, Germany) and acetate was measured by an acetate test kit (R-Biopharm).

### Fed-batch culture with EnBase^® ^Flo

#### Medium composition

EnBase^® ^Flo mineral salt medium was applied from BioSilta Oy (Oulu, Finland). The medium was a fully liquid formulation basically composed according to Panula-Perälä et al. [19] with small modifications. Especially the medium contained an improved trace element composition according to Soini et al. [20]. Additionally in some of the cultures boosting with complex additives was performed with the EnBase^® ^Booster from BioSilta, consisting of a mixture of peptone and yeast extract, as earlier described [18].

#### Fed-batch culture

Precultures were grown overnight in EnBase^® ^Flo and grew to OD_600 _of 10 to 15. The cultures were centrifuged at 13000 × g for 5 min and the pellet was then resolved in fresh EnBase^® ^Flo medium. Main cultures were inoculated with an OD_600 _of 0.15.

EnBase^® ^Flo cultures were performed in a BIOSTAT^® ^CultiBag RM 20 optical system using a CultiBag RM 2 L bag with a culture volume of 1 L. The rocking angle was set to the maximum tilt level of 10°, the rocking rate was also set to a maximum of 42 rocks min^-1^, the temperature and air flow rate were set to 30°C and 1 L min^-1^, respectively.

The glucose delivery rate was adjusted by the concentration of amylase (BioSilta Oy, Oulu, Finland) added to the medium. The amylase concentrations used during the cultures varied from 1.5 to 6 U L^-1^. ADH expression was induced at OD_600 _of 14 to 17 by addition of 1 mM IPTG.

#### Analytical methods

##### Cell growth

Growth of the cultures was followed by measuring the light absorbance at 600 nm (OD_600_), by measuring the cell wet weight, and by determination of the cell dry weight. Therefore 1 ml cell suspension was centrifuged in pre-dried and pre-weighed 1 ml test tubes at 13000 × g for 5 min. After removal of the supernatant, the samples were measured for cell wet weight and then dried at 80°C for at least 24 h. An OD_600 _of 1 resulted in a cell wet weight of 1.7 g L^-1 ^and 0.39 g L^-1 ^of dry weight.

##### Exhaust gas analysis

Oxygen in the exhaust gas was analyzed with a Maihak Oxygor 6 N, carbon dioxide was analyzed with a Maihak Unor 6 N (both Sick Maihak GmbH, Reute, Germany). The oxygen consumption and carbon dioxide production were derived based on a mass balance of the gas phase as shown in eq. 1 for the volumetric oxygen consumption and in eq. 2 for the volumetric carbon dioxide production rate in mol L^-1 ^h^-1^.(1)(2)

In eq. 1 and 2, *V*_*F  *_represents the liquid (working) volume in L,  the gas flow rate at the inlet in nL h^-1^, , , and  the fraction of O_2_, N_2_, and CO_2 _in the inlet gas stream, , , and  the fraction of O_2_, N_2_, and CO_2 _in the off gas stream, and *V*_*M *_the molar volume of 22.4 L mol^-1^.

The volumetric oxygen transfer coefficient (*k_L_a*) was determined by the application of the quasi-steady state method [23].

##### Glucose and Polysaccharide content

Measurements of glucose content were performed with the glucose hexokinase FS measuring kit (DiaSys Diagnostic Systems GmbH, Holzheim, Germany). To measure the polysaccharide content an acid hydrolysis was performed. Therefore 100 μl of sample were mixed with 100 μl of 2 N HCL and heated to 100°C for 2 hours. The neutralization was done with 100 μl 2 N NaOH and 100 μl 0.5 M Sörensen buffer. After neutralization, glucose concentration was measured enzymatically in the polysaccharide samples.

##### Analysis of medium components

Acetate, lactate and ethanol were analyzed using an HPLC 1200 Liquid Chromatography System (Agilent Technologies Inc., Santa Clara, CA) equipped with a refractive index detector (RID). As column, a HyperRez XP Carbohydrate H+ (300 × 7.7 mm, 8 μm, Fisher Scientific Inc.) was applied. 5 mM sulfuric acid was used as a running buffer. Peaks were integrated with the software package Chemstation Version Rev. B.04.01 (Agilent). Prior to analysis, culture samples of *E. coli *were centrifuged for 5 min at 13000 × g and 4°C and afterward filtrated (0.2 μm Nylon, Carl Roth GmbH, Karlsruhe, Germany).

##### Protein analysis

Soluble and insoluble protein of the cells were isolated and analyzed with SDS-PAGE. Therefore, a cell pellet corresponding to an OD_600 _of 10 was resuspended in 0.1 M Tris/HCL (1 mM EDTA, pH 7), and 2 μl of lysozyme (50 mg ml^-1^) were added. Cells were incubated on ice for 30 min. Afterwards, cells were sonicated (UP200, Dr. Hielscher GmbH, Teltow, Germany) on ice 3 times for 30 sec (45 sec off) with an amplitude of 70% and with a sonotrode of a diameter of 1 mm. Soluble protein was harvested from the supernatant by centrifugation (5 min, 13000 × g, 4°C). The pellet was resuspended in 1 ml of 0.1 M Tris/HCL and analyzed for insoluble protein. The samples were loaded onto 12% polyacrylamide gels. Staining was performed with Coomassie Brilliant Blue R 250.

## Results

### Batch cultivation

**Figure 1:**
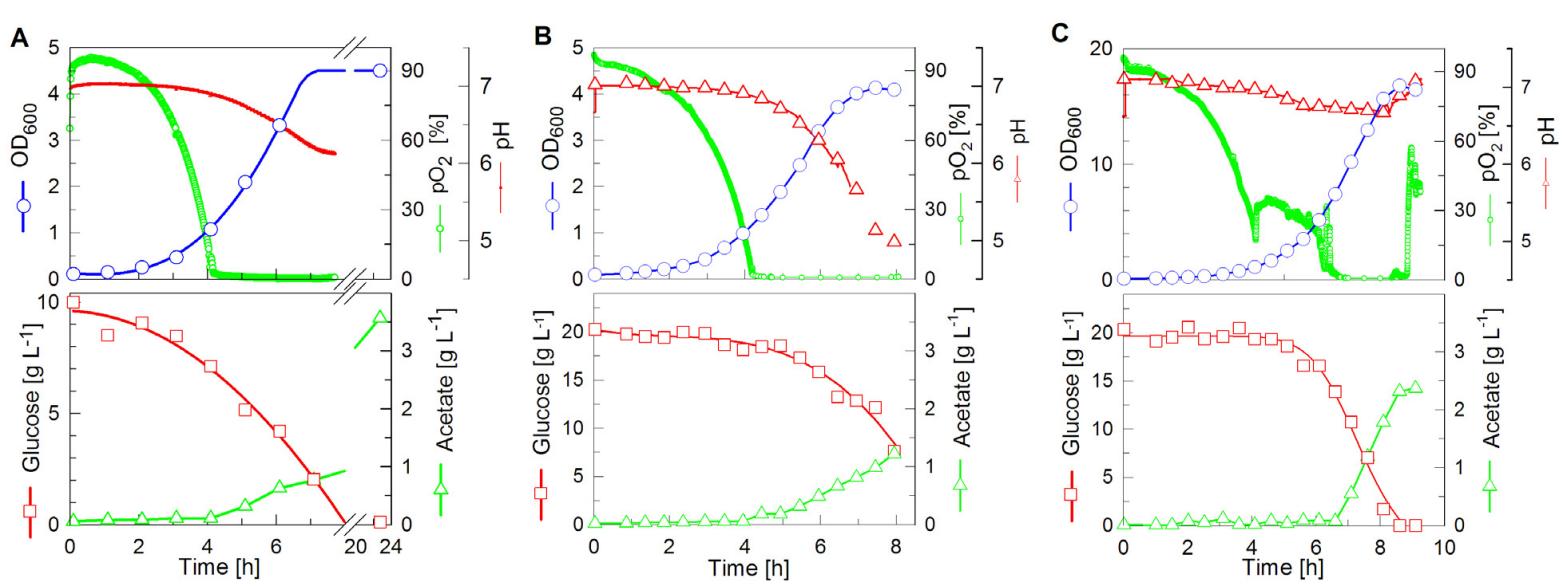
**Batch cultivation of *E. coli *BL21 in glucose mineral salt medium in (A) a shake flask with the Sensolux monitoring system and (B, C) with the CultiBag RM system**. Cultivation conditions: 10 L bag with 5 L working volume, maximum rocking angle, 35 rocks per min, 37°C, air flow rate 1 L min^-1 ^in (B). In (C) the air flow rate was 1 L min^-1 ^at the start of the cultivation. After the pO_2 _decreased to 50% a dual controller mode was started to maintain the pO_2 _by (i) increasing the air flow rate to 6 L min^-1 ^and (ii) pulse addition of pure oxygen into the inlet air stream (up to 100%).

The first aim of this study is the comparison of the growth rate of an *E. coli *BL21(DE3) strain, which was grown in the CultiBag RM system in minimal medium, to that of a culture grown in a shake flask under similar conditions (Fig. 1A-C). Therefore, batch cultivations were conducted at 37°C and 200 rpm in shake flasks, or with the maximum rocking angle and speed in the CultiBag RM system.

For batch cultivation *E. coli *BL21(DE3) was cultivated in the CultiBag RM system on mineral salt medium with 20 g L^-1 ^glucose as the sole carbon/energy source (Fig. 1B,C). The culture reached an OD_600 _of 4.1 after 7 hours and grew with a maximal specific growth rate (μ_max_) of 0.74 h^-1^. The obtained final cell density (OD_600 _= 4.1, see Fig. 1B) was similar to values obtained in shake flasks (OD_600 _= 4.5, Fig. 1A). The biomass yield on glucose (Y_X/S_) was only approximately 0.16 (g cell dry weight per g glucose). This was in the range of the value also calculated for the shake flasks (Y_X/S _= 0.18, Table 1). Based on the measured time courses of the dissolved oxygen concentration in the shake flask and CultiBag RM cultures, a likely reason for the low yield was the apparent oxygen limitation in both systems (see Fig. 1A,B). The CultiBag RM system offers the possibility of pulsing pure oxygen to the culture. The improved oxygen transfer should thus lead to higher cell densities. In Fig. 1C it is shown that in a batch culture with oxygen pulsing OD_600 _of 18 can be reached at 37°C which corresponds to a yield coefficient of Y_X/S _= 0.35. Oxygen limitation in this case was observed at OD_600 _of about 7 compared to 1 without extra oxygen supply.

**Table 1:** Biomass yield on glucose (Y_x/s_) for the cultivations performed in this study.

Medium/strain	Biomass yield, Y_x/s _[g g^-1^]
Batch cultivation, BL21(DE3), shake flask	0.18
Batch cultivation, BL21(DE3), CultiBag RM	0.16
Batch cultivation with oxygen sparging, BL21(DE3), CultiBag RM	0.35
Fed-batch cultivation, DO-stat principle, BL21(DE3)	0.52
EnBase^® ^(glucose limited fed-batch) and boosting, BL21	0.49
Fed-batch with EnBase^®^, RB791 pADH	0.52
Fed-batch with EnBase^® ^and boosting, RB791 pADH	0.56

### Fed-batch cultivation is feasible in the CultiBag RM system

**Figure 2:**
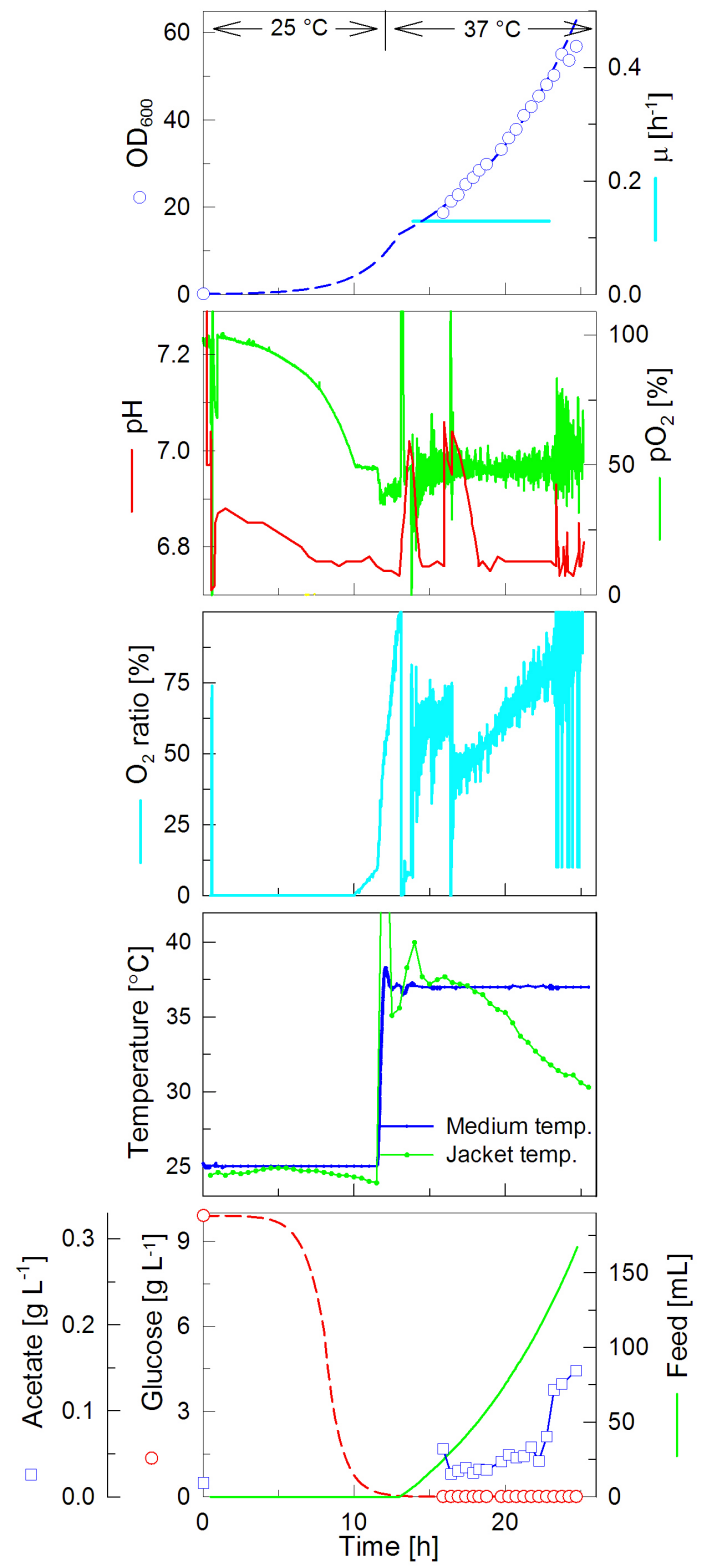
**DO-stat based Fed-batch cultivation of *E. coli BL21 *in minimal medium with the CultiBag RM system**. Cultivation conditions: 10 L bag with 4 L filling volume, maximum rocking angle, 35 rocks min^-1 ^during the batch phase and the maximum rocking speed (42 rocks min^-1^) during the feeding phase, cultivation temperature 25°C during the batch phase and increase to 37°C after 11 hours. The pO_2 _was controlled to 50% by a dual control of (i) the gas flow rate from 1 to 6 L min^-1 ^and (ii) pulsing of pure oxygen into the inlet air stream (up to 100%, maximum total flow rate of 6 L min^-1^, named O_2 _ratio).

To increase the volumetric cell yield a glucose-limited fed-batch cultivation mode was applied. Therefore, a 10 L CultiBag RM equipped with optical sensors for pH and pO_2_, was filled with 4 L of cultivation medium. An initial glucose concentration of only 10 g L^-1 ^secured that the oxygen transfer rate was sufficient to support fully aerobic growth during the initial batch phase. After 13.5 hours, when the initial glucose was exhausted, continuous feeding of a highly concentrated glucose solution with a constant rate was started (Fig. 2). The feeding rate was exponentially increased over the time to guarantee a specific growth rate of 0.13 h^-1^. Consequently also the partial pressure of oxygen (pO_2_) decreased exponentially. When the pO_2 _reached the set point of 50%, a dual pO_2 _controller was started to maintain this level. Firstly, the air flow rate was increased to a maximum of 6 L min^-1^, and secondly the oxygen content was stepwise increased by pulses of pure oxygen to a maximum of 100% of the total gas flow rate. With this procedure, the culture grew to an optical density of OD_600 _= 58 at 37°C with a very small volume increase only (app. 150 ml, < 5%). An advantage of the CultiBag RM system is the reliable temperature control, also at higher cell densities when the metabolic rates are high. Fig. 2 demonstrates that the temperature could be maintained over the whole cultivation close to the setpoint.

### High cell densities with enzyme based glucose delivery

**Figure 3:**
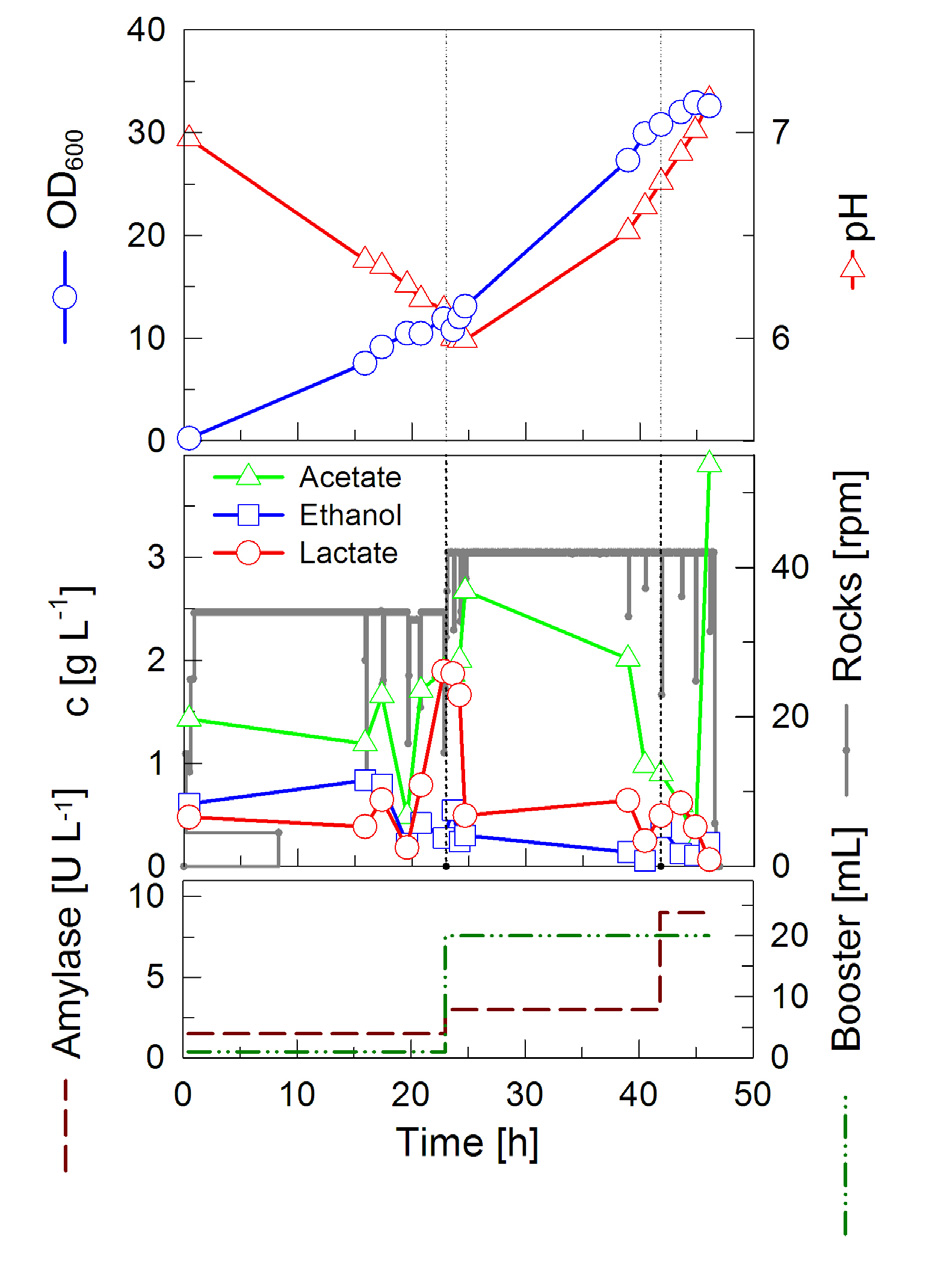
**Cultivation of *E. coli *BL21 in EnBase^® ^Flo with the CultiBag RM system**. The cultivation was performed in a 2 L bag with 1 L EnBase^® ^Flo medium containing an amylase concentration of 1.5 U L^-1^, which was increased up to 9 U L^-1^. Boosting solution was added once after 23 hours.

In most processes the aim is to reach highest possible cell densities in a short time under aerobic conditions. With EnBase^® ^Flo an optimal enzyme concentration can be found by stepwise increasing its concentration. This is exemplary shown in Fig. 3. Such an optimization can be even performed if there are no online pO_2 _or pH monitoring systems available. Additionally the following experiments were performed without pH control, because most rocking-motion-systems do not contain the possibility for on-line measurement and control. The use of bags which are equipped with such sensors is more expensive. Aside from increasing the biocatalyst concentration also the effect of additives, such as the EnBase^® ^Booster from BioSilta, can be tested, as it was done in the cultivation in Fig. 3 after 23 hours. Since the addition of boosting substrate may provoke an increase in pH, biocatalyst was added together with the boosting solution to balance the pH as proposed in [18] to inhibit the catabolic use of amino acids as carbon/energy source. The initial biocatalyst concentration of 1.5 U L^-1 ^was increased to 3 U L^-1^. At the end of the cultivation, a further biocatalyst pulse was performed with 6 U L^-1 ^to evaluate whether there is further potential in increasing the substrate release and enhancing the growth rate. Indeed, the first addition of biocatalyst and boosting solution led to an increase in the growth rate. A final optical density of OD_600 _= 33 was reached (Fig. 3).

Generally, the results clearly demonstrate that EnBase^® ^Flo provides benefits in the CultiBag RM system, as its application leads to much higher biomass concentration compared to the batch mode, while only the enzyme and the boosting solution are added.

### Recombinant protein expression

Another aim of this study was to examine whether the enzyme based glucose delivery is also applicable for recombinant protein production in rocking-motion-type bioreactors. Although generally the fed-batch procedure has been extensively applied for successful production of many proteins, the challenge was to develop a strategy which is robust enough so that it also can be used without any sensors or further control. Despite this aim, in some of the cultures, pH and DO sensors were applied to evaluate the conditions provoked by the additives. As an example for a recombinant production system *E. coli *RB791 pAdh was used, which expresses a recombinant alcohol dehydrogenase very well in fed-batch cultivation and in Enbase^® ^Flo deep-well plate cultures [18,21].

Due to the application of the pO_2 _sensor, the *k_L_a *value of the system could be estimated during the growth phase of the microorganisms. The mean value at measured time points was 55 h^-1 ^(standard deviation: 6.6). This is in the same order of *k_L_a *values described in literature for rocking-motion-type bioreactors as mentioned in the introduction of this report. The oxygen transfer rate in the rocking-motion-type bioreactor is approximately 6 fold lower compared to the *k_L_a *in stirred tank bioreactors of the same volume and appropriate settings for *E. coli *cultivation in our lab (data not shown).

**Figure 4:**
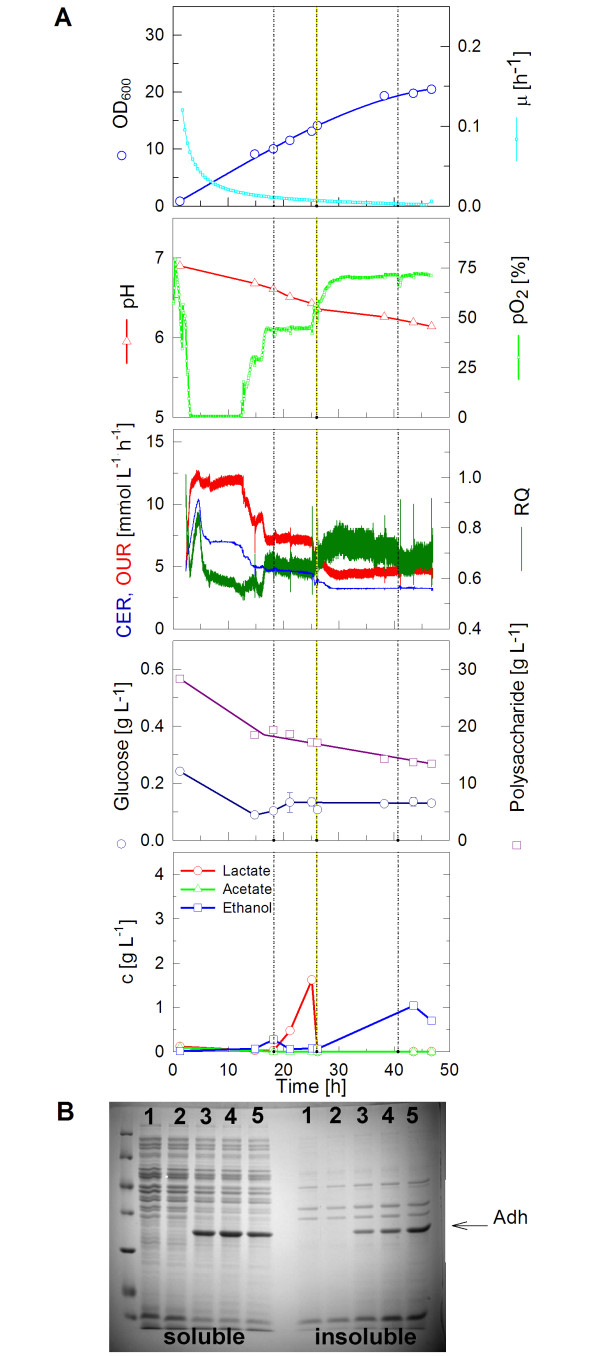
**Cultivation of *E. coli *RB791 pAdh in EnBase^® ^Flo with the CultiBag RM system**. (A) Data of a cultivation in a 2 L bag with 1 L EnBase^® ^Flo medium containing 1.5 U L^-1 ^of amylase. Trace elements and MgSO_4 _were added after 18 and 41 hours and ammonium sulfate was added after 26 hours. Protein expression was induced by 1 mM IPTG after 26 hours. (B) Coomassie Brilliant Blue stained SDS polyacrylamide gel with soluble (left lanes) and insoluble protein samples (right lanes). Same amounts of cells were loaded onto each lane. Lane 1) before induction; 2) at induction; 3) after 12 h; 4) after 17 h; 5) after 20 h. Most left lane - size standard.

To observe the growth behavior of this *E. coli *strain under non enhanced conditions and to get a starting point for further optimizations, control cultivations were performed in the EnBase^® ^Flo cultivation medium without any boosting (see Fig. 4). The medium contained 1.5 U L^-1 ^of biocatalyst from the beginning. Only ammonium sulfate was added once (after 26 hours), trace elements and MgSO_4 _twice (after 18 and 41 hours) during the course of the cultivation to obtain higher cell densities. The culture grew almost linearly and reached an OD_600 _of 21 after 47 hours (Fig. 4A). Even after induction (after 26 hours), biomass growth did not decelerate markedly. It is remarkable that a high Y_X/S _was obtained despite the rapid decrease of the pO_2 _concentration to depletion in the beginning of the cultivation (see Table 1). The cultivation turned into glucose limitation after 12 h, indicated by the increase of the pO_2_. This increase was not as rapid as it can be seen in standard batch cultures for two reasons: (i) there is a continuous glucose release through the amylase in the medium, and (ii) we know from other cultures that acetate is formed during the initial growth phase due to a high release/consumption rate for glucose. This acetate is consumed when the culture runs into glucose limitation, i.e low release/consumption rate due to the increased cell density. After this phase (at about 17 hours) the DO level remained stable (after 19 h). In the first 17 h of cultivation the mean respiratory quotient remained around 0.6, before it increased to around 0.7. The volumetric oxygen consumption and the volumetric carbon dioxide production rates were both low at this experiment. Mean values were determined to be 7.3 mmol L^-1 ^h^-1 ^and 4.7 mmol L^-1 ^h^-1^, respectively.

Interestingly, in the time between 20 and 25 h, lactate but not acetate increased. This observation we already made earlier when an optimized trace element solution supplied with nickel, molybdenum and selenium was applied [20]. Such a lactate accumulation profile was a typical indicator of oxygen limitation. Thus, despite the sensor signal showing of 40% saturation of pO_2_, this lactate accumulation suggests oxygen limitation, which might be due to mixing inhomogeneities of the pO_2 _in the bag.

Later, addition of IPTG for induction of the alcohol dehydrogenase led to a decrease of respiration. Consequently, the pO_2 _level rose and during this phase also ethanol increased to 1 g L^-1^. During the cultivation, by consumption of 15 g L^-1 ^polysaccharide, a cell dry weight of 7.8 g L^-1 ^was produced, corresponding to a yield of biomass per substrate Y_X/S _of 0.52 g g^-1^. This is in good agreement with data which have been obtained with glucose mineral salt medium fed-batch cultures of related strains earlier [20,24].

The expression of the ADH was studied by analyzing soluble and insoluble protein fractions of the cells by SDS-PAGE (Fig. 4B). The ADH product is clearly visible after induction. At 12 hours after induction, the biggest portion of the produced protein is in soluble form. Prolongation of the cultivation caused a decrease of the ratio of soluble to insoluble product. A similar behavior of ADH has been seen before (see [18]).

**Figure 5:**
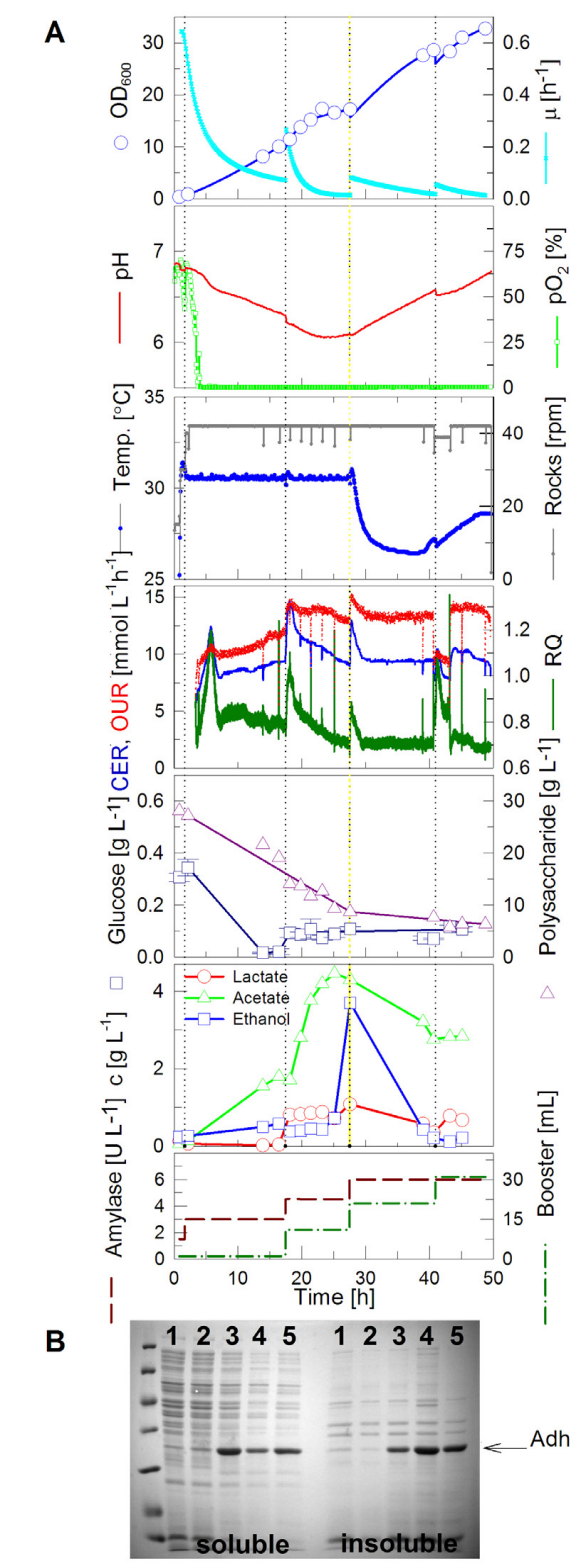
**Cultivation of *E. coli *RB791 pAdh expressing a heterologous alcohol dehydrogenase in the CultiBag RM system with 1 L of EnBase^® ^Flo medium with the addition of complex additives (EnBase^® ^Booster)**. (A) In this cultivation the amylase concentration was stepwise increased from 1.5 to 6 U L^-1^; the boosting solution was added three times. Protein expression was induced with 1 mM IPTG after 27 hours. (B) Coomassie Brilliant Blue stained 12% SDS polyacrylamide gel of soluble and insoluble protein samples. Same amounts of cells were applied to each lane. Lane 1) before induction; 2) at induction; 3) 11 h; 4) 16 h; 5) 21 h after induction.

Although this experiment showed that recombinant ADH is well produced in the BIOSTAT^® ^CultiBag RM system and the process is very similar to cultures performed in deep wells (data not shown, but earlier published in [18]), it was further optimized. We considered to increase the cell density and thus the volumetric yield by providing extra ammonia nitrogen and amino acids to the culture. This should keep the pH at a level which is well tolerated by *E. coli *as discussed before. Therefore, in the following cultivation (Fig. 5), EnBase^® ^Booster solution was added together with the biocatalyst at three time points. This type of cultivation yielded an OD_600 _of 33 after 49 hours. At this time, the culture still had not reached its maximum optical density and continued linear growth. Besides the boosting solution, amylase was added, so that it reached a final concentration of 6 U L^-1^. Every time the solutions were added, the culture resumed to grow faster again. Still, the glucose concentration in the medium remained low. It is obvious that after 25 hours the speed of degradation of the polysaccharide and the glucose supply rate slowed down. This correlates well with the increase in pH, indicating that the metabolism of the microorganisms is changing and amino acids are now degraded to generate energy (see also [18]).

It is remarkable that the culture grew under oxygen limiting conditions over the whole cultivation period, being a further indication of the usefulness of supplying a mineral salt growth medium with the additional trace elements (Mo, Ni, Se) which promote growth under oxygen limitation [20]. The respiratory activity was enhanced compared to the control experiment with *E. coli *RB791 pAdh. The mean value of the respiratory quotient was restored back to values obtained in *E. coli *BL21 cultivation with EnBase^® ^Booster (0.79). Before 20 h of cultivation, the respiratory quotient remained above 0.8, and dropped to 0.7 in the following. The mean volumetric oxygen consumption rate and carbon dioxide production rate was determined to be 12.3 mmol L^-1^h^-1 ^and 9.6 mmol L^-1^h^-1^, respectively. That is significantly higher compared to the control cultivation with *E. coli *RB791 pAdh without boosting. The *k_L_a *value determined during the growth phase in this experiment was identical to the one obtained in the control experiment (data not shown).

When analyzing the protein content of the cells, a result similar to the first experiment was obtained (Fig. 5B). The ADH was overexpressed strongly. Also in agreement with the earlier results, a greater portion of the soluble form was present in the cells at early samples, whereas after 21 hours more protein became insoluble.

The extra additions of complex additives and ammonia had a positive outcome and resulted in a well growing culture up to an OD_600 _of 33. The expression of recombinant protein appeared similar compared to earlier studies (cf. [18]).

## Discussion

The successful establishment of high cell density cultivation of microorganisms in a disposable bioreactor opens new application dimensions for disposable cultivation systems. The aim of this work was to evaluate the disposable rocking-motion-type cultivation system for microbial high cell density cultivation.

The high cell density cultivation in CultiBag RM was performed under fed-batch conditions, which represents the most common technique for achieving a high specific productivity by avoiding oxygen limitation. During the fed-batch process, it is critical to control the specific growth rate to avoid the formation of inhibitory by-products.

It was proven in this study, at the example of the widely used strain *E. coli *BL21 that the feed control system of the CultiBag RM can be used to grow the culture to reasonable high cell densities by a standard glucose limiting fed-batch mode. Thereby a cell density of OD_600 _= 58 (which corresponds to a biomass concentration of 22 g L^-1^) could be achieved after 20 h of cultivation. The obtained amount of biomass was 13 times higher than in batch cultivations that were performed in disposable rocking-motion-type bioreactors and in a shake flask control experiments and even tree times higher compared to a batch culture with oxygen sparging.

In the second part of the paper we investigate the applicability of the Enbase^® ^Flo system in the Cultibag RM system. Enbase^® ^Flo is an internal substrate delivery system, where by the use of a glucoamylase glucose is slowly released from a soluble starch derivative [18]. Recently the medium and the procedure for recombinant protein production was optimized to make the system independent on addition of pH control agents, which may display an advantage in some disposable systems, and even save cost to the user of the CultiBag R system.

In the cultivations with the EnBase^® ^Flo system initially *E. coli *BL 21 was used to estimate what maximal biomass concentration could be reached in rocking-motion-type bioreactors. The maximal optical density reached was OD_600 _= 33 (corresponding to 13 g L^-1^), the typical cell density for EnBase^® ^Flo cultivation in deepwell plates and shake flasks (cf. [18]).

As an example for a recombinant protein production process, the first cultivation with the recombinant strain *E. coli *RB791 pAdh was performed under the same conditions as with the wild type strain, but without additional supply of complex nutrients (called control cultivation). The optical density reached in the first cultivation was less than that obtained with the wild type strain and EnBase^® ^Booster. Thus, the positive effect of the addition of complex nutrients during the cultivation was demonstrated in a next experiment. This improving effect of EnBase^® ^Booster on the growth and recombinant protein expression has been investigated in the literature before [18,25].

In the control cultivation with *E. coli *RB791 pAdh (Fig. 4), the concentration of glucose could be kept low (below 0.2 g L^-1^) throughout the cultivation. Therefore, it was investigated whether the higher concentration of biocatalyst can be added to the cultivation medium to increase the growth rate while still keeping the process under glucose-limited conditions. It could be observed (Fig. 5) that the repeated addition of amylase caused acetate accumulation in the medium. The addition of EnBase^® ^Booster before inducing of recombinant protein also increased acetate accumulation to 4 g L^-1^. However, based on the exhaust gas compartment, no negative effects on the metabolic activity of the microorganisms could be detected. In contrast, the complex additives clearly enhanced the respiratory activity, limitations were reduced. Since the respiratory quotient was increased each time, boosting solution affected anaerobic pathways or pathways with a net surplus of carbon dioxide production. Hence, it can be assumed that these kinds of reactions are not related to the oxygen transfer rate.

Comparing the protein content (Figs. 4B and 5B) with and without the addition of complex nutrients at the inducing time, it could be observed that the addition of EnBase^® ^Booster increases the amount of soluble protein. It was also observed that the addition of EnBase^® ^Booster 21 hours after the induction of protein decreases the amount of soluble protein and equally increases the amount of insoluble protein. Therefore in case of Adh early harvesting of the culture would be proposed.

All cultivations with EnBase^® ^Flo were performed without oxygen control to show the applicability of the method also for simple rocking-motion systems. During the course of cultivations, the oxygen partial pressure dropped to zero because of the limited oxygen transfer by headspace aeration. Despite the low k_L_a values of 50 to 55 h^-1 ^determined in the experiments, the culture continued to grow linearly for several hours. Studying the polysaccharide content over the time, it could be seen that the polysaccharide concentration decreased rapidly during the cultivation. After 25 h, the speed of degradation of the polysaccharide was diminished. In the end of the cultivation about 20% of the polysaccharide remained still in the medium. This correlated well with the increase in pH due to acetate uptake by the microorganism.

## Conclusions

This study is to our knowledge the first investigation issuing the use of fed-batch technology for *E. coli *cultivation and recombinant protein production for rocking-motion-type bioreactors. The study indicates that there is a potential of using rocking-motion-type systems which are limited by a low oxygen transfer rate for elevated biomass production. In view of its control system the CultiBag RM system provides the general features which are typical for standard bioreactors. This allows also the application of more advanced control strategies, such as exponential feeding procedures and feed-back control of the pO_2_. Pulsing of extra oxygen provides some advantage in overcoming the low oxygen transfer rates.

A wide interest for using rocking-motion-type bioreactors is especially expected in facilities which do not have simple access to, or experience with, bioreactor facilities. Enbase^® ^Flo provides a simple alternative for a high cell density type of cultivation without the need for the user to be a specialist in fermentation technology. In this system the amount of an enzyme determines the glucose release rate and all optimisation can be done at the microwell or deepwell plate stage. Here we show at the example of ADH that such preoptimised processes simply can be transferred to a rocking-motion-system, without the need for setting up any extra control. This would provide a simple scale up to the 100 scale. On top of this, further modifications can be done for improving the volumetric yield further, e.g. by combining an initial Enbase^® ^Flo culture with a subsequent fed-batch of glucose as an external feed, or providing further complex additives as shown in the culture of Fig. 5. Aside from the principal feasibility of Enbase^® ^Flo in a rocking-motion-type bioreactor which we demonstrated here, it will be interesting to see whether there is a direct scalability of such a process directly from deepwell plates to the scale of rocking-motion-type systems in a similar way as it was demonstrated recently for the scale up to a stirred tank bioreactor process (see [16,26]).

## Competing interests

The authors declare that they have no competing interests.

## Authors' contributions

TD performed the batch and fed-batch cultures. EMM, JG and DS carried out the cultures with EnBase^® ^and did the analyses. EMM and JG drafted the manuscript. DS participated in the cultivations and the analysis of the samples. SJ helped at the sample analysis by HPLC and the exhaust gas analysis. GG and TA participated in the planning of the study; they were responsible for the batch and fed-batch experiments, and they participated in the finalization of the manuscript.

PN supervised the study, and participated in its design and coordination and helped to draft the manuscript. All authors read and approved the final manuscript.
